# Caffeic Acid Attenuates Diabetic Kidney Disease via Modulation of Autophagy in a High-Fat Diet/Streptozotocin- Induced Diabetic Rat

**DOI:** 10.1038/s41598-017-02320-z

**Published:** 2017-05-23

**Authors:** Marwa Matboli, Sanaa Eissa, Doaa Ibrahim, Marwa G. A. Hegazy, Shalabia S. Imam, Eman K. Habib

**Affiliations:** 1 Medical Biochemistry and Molecular biology Department, Faculty of Medicine, Ain Shams University, P.O. box 11381, Abbassia, Cairo, Egypt; 2 Pharmacology Department, Faculty of Medicine, Ain Shams University, P.O. box 11381, Abbassia, Cairo, Egypt; 3 Biochemistry Department, Faculty of Science, Ain Shams University, P.O. box 11381, Abbassia, Cairo, Egypt; 4 Phytochemistry Department, National Desert Research Center, Cairo, Egypt; 5 Anatomy & Embryology Department, Faculty of Science, Ain Shams University, P.O. box 11381, Abbassia, Cairo, Egypt

## Abstract

The aim of this study is to evaluate the anti-diabetic nephropathy effect of Caffeic acid and to prove our hypothesis for its mechanism of action that it may occur by reactivation of autophagy pathway via suppression of autophagy regulatory miRNAs. In vivo, high-fat diet and streptozotocin-induced (HFD-STZ) diabetic rats were treated with Caffeic acid once per day for 12 weeks before and after development of diabetic nephropathy. Blood and urine biochemical parameters, autophagy transcripts and their epigenetic regulators together with renal tissue morphology were investigated. In diabetic rats, Caffeic acid intake, caused improvement in albumin excretion,blood glucose, reduced renal mesangial matrix extension with increased vacuolation and reappearance of autophagosomes. Meanwhile, it resulted in autophagy genes up-regulation [RB 1-inducible coiled coil protein (RB1CC1), Microtubule-associated proteins 1A/1B light chain 3(MAP1LC3B), Autophagy related gene (ATG-12),] with simultaneous reduction in their epigenetic regulators; miRNA-133b, −342 and 30a, respectively. These above mentioned effects were more significant in the diabetic nephropathy Caffeic treated rats than in the prophylactic group. Based on our results we postulated that caffeic acid modulates autophagy pathway through inhibition of autophagy regulatory miRNAs, that could explain its curative properties against diabetic kidney disease.

## Introduction

The prevalence of diabetic kidney disease (DKD) has been increasing world wide. Therapeutic strategies, including antidiabetic drugs and inhibitors of the renin– angiotensin, can postponed DKD. Accordingly, we need to look for a possible remedial target to treat or avoid DKD^1^.

Recent studies highlighted the role of genetic and epigenetic mechanisms in the regulation of autophagy process as well as the pathogenesis of DKD^2^. Reduction of autophagy results in oxidative stress, podocyte injury, mesangial cell proliferation, glomerular endothelial dysfunction, accumulated collagen and TGF-β1^3^. Thus, a cytoprotective multitarget modulation of autophagy is significantly required to attenuate renal damage in diabetes^4^. Specific miRNAs have currently been identified as significant epigenetic modifiers of autophagy linked genes. In fact, these autophagy linked genes’ mRNA includes, the target sequence for miRNAs related to diverse families^5, 6^. The gene networks regulating autophagy pathway were determined using a system biology and unrevealed miR-130, miR-98, miR-124, miR-204, and miR-142 as presumed posttranscriptional modulators of this pathway at different levels^6^. Therefore, unraveling the significance of autophagy-miRNA interaction in DKD might lead the way to novel diagnostic and molecular therapeutic targets for DKD^7^. We have therefore focused on a strategy for the resumption or activation of autophagy. Proautophagic drugs are a promising class of compounds for diabetic nephropathy regression. Recent studies revealed many phytochemical constituents that can induce autophagy^8^.

Wang and his colleagues reported that caffeic acid extract of *Artemisia dracunculus L*. enhances insulin receptor signaling and modulates gene expression in skeletal muscle in KK-Ay mice^9^. Scientists recently reported that a semisynthetic compound derived from caffeic acid derivative induces DNA damage and apoptosis in tumor cells via induction of autophagy in cancer cells^10^. Also, Caffeic acid derivative have been reported to have a protective role in renal damage^11^. We sought to determine the efficacy of caffeic acid on modulating autophagy pathway via inhibiting expression of miRNA related DKD with subsequent upregulation of its target autophagy related genes utilizing HFD-STZ -diabetic rats.

The rational of this study was based on; (1) Bioinformatics analysis to retrieve a set of 3 DKD-characterestic autophagy genes(RB 1-inducible coiled coil protein (RB1CC1), Microtubule-associated proteins 1A/1B light chain 3(MAP1LC3B), Autophagy related gene (ATG-12)]. Then to choose miRNAs (miR-133b, miR-342 and miR-30a) relevant to DKD and acting as epigenetic regulators of the former autophagy genes based on previous microarray studies; (2) Experimental validation to characterize the efficacy of caffeic acid on modulating the expression of chosen miRNA-autophagy target gene pairs in HFD-STZ induced rat model.

## Results

### Effect of caffeic acid on fasting blood glucose and other metabolic parameters in HFD-STZ induced diabetic rats within the first five weeks

**Figure 1:**
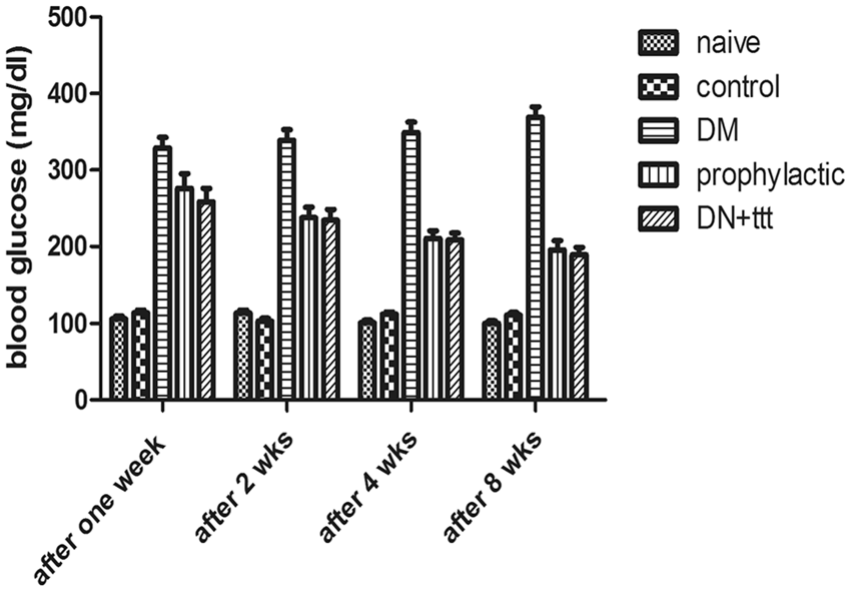
The development of HFD-STZ -induced diabetic nephropathy in rats. Change in blood glucose concentrations. Two way repeated measure ANOVA, followed by Bonferroni’s post-tests, N = 8/group.

STZ resulted in a significant increase in blood glucose level, compared to either control or naïve group. [F = 463.8, p < 0.01], with a significant increase of blood glucose level over time [F = 319.7, p < 0.01]. Bonferroni posttest was used to compare the both groups at different time points. Blood glucose level in HFD-STZ treated group was significantly higher from the naïve group starting at week 2, and progressively increased till week 4 (P < 0.01; Fig. 1).

After 12 weeks, the levels of FBG, serum total cholesterol, LDL, total triglycerides in the DM group were significantly higher than those of the NC group. Compared with DM group, Caffeic acid treatment and prophylactic groups showed marked lowering of FBG and lipid profile indicating a clear improvement in the glucose and lipid metabolism (Table 1).

**Table 1:** Effect of Caffeic acid on urine and serum metabolic and renal markers.

	Mean	Std. Error	F	Significance
**Serum creatinine concentration (mg/dl)**				
control	1.00	0.000	7.070	0.0001
placebo	1.00^a^	0.000		
diabetes mellitus	2.00	0.000		
Prophylactic	2.00	0.000		
Diabetic nephropathy	1.83	0.167		
STZ+ Caffeic acid	1.43	0.202		
**Urea (mg/dl)**				
control	38.00	5.000	72.806	0.0001
placebo	33.50^a,b^	2.500		
diabetes mellitus	57.33	2.231^b^		
Prophylactic	84.29	3.107^b^		
Diabetic nephropathy	81.67	1.498		
STZ+ Caffeic acid	30.14	2.857^b^		
**BUN (mg/dl)**				
control	17.50	2.500^b^	71.657	0.0001
placebo	15.50^a^	1.500^b^		
diabetes mellitus	26.67	1.085^b^		
Prophylactic	39.29	1.392^b^		
Diabetic nephropathy	38.17	0.703		
STZ+ Caffeic acid	14.14	1.370^b^		
**HDL-cholesterol (mg/dl)**				
control	34.50	2.500	3.750	0.550
placebo	31.00	1.000		
diabetes mellitus	33.17	2.798		
porphylactic	28.71	1.443^b^		
Diabetic nephropathy	28.17^a^	1.600		
STZ+ Caffeic acid	25.86	1.010^b^		
**Triglycerides (mg/dl)**				
control	134.00	7.000^b^	20.844	0.0001
placebo	122.00	3.000^b^		
diabetes mellitus	180.83^a^	6.735^b^		
Prophylactic	50.71^a^	7.677^b^		
Diabetic nephropathy	51.83	17.252		
STZ+ Caffeic acid	129.00	11.093^b^		
**LDL-cholesterol (mg/dl)**				
control	20.00	14.000	2.786	0.040
placebo	28.00	5.000		
diabetes mellitus	11.33	11.910		
Prophylactic	61.71	12.124		
Diabetic nephropathy	26.33	7.186		
STZ+ Caffeic acid	30.29	9.203		
**Total Cholesterol (mg/dl)**				
control	81.50	12.500	1.109	0.382
placebo	83.50	6.500		
diabetes mellitus	81.00	10.954		
Prophylactic	100.43	11.193		
Diabetic nephropathy	78.00	8.714		
STZ+ Caffeic acid	71.71	7.402		
**Urine creatinine Concentration (mg/dl)**				
control	59.00	1.000	24.659	0.0001
placebo	55.50	1.500		
diabetes mellitus	38.17	2.574		
porphylactic	115.71	11.047		
Diabetic nephropathy	33.17	2.676		
STZ+ Caffeic acid	60.43	0.948^b^		
**Urine volume (liter/day)﻿**				
control	4.50	0.500^b^	138.861	0.0001
placebo	4.00^a^	0.000^b^		
diabetes mellitus	13.50	0.428^b^		
Prophylactic	17.71^a^	0.747^b^		
Diabetic nephropathy	18.50	0.563		
STZ+ Caffeic acid	4.00	0.218^b^		
**Creatinine clearance (ml/min)**				
control	0.1607	0.02495	33.400	0.0001
placebo	0.1172	0.01275		
diabetes mellitus	0.1673^a^	0.01508		
Prophylactic	0.6620	0.06468^b^		
Diabetic nephropathy	0.2359	0.01889		
STZ+ Caffeic acid	0.1148	0.0093^b^		
**Albuminuria**				
control	16.00	0.000^b^	135.014	0.0001
placebo	16.00^a^	0.000^b^		
diabetes mellitus	28.17^a^	0.872^b^		
Prophylactic	31.57^a^	0.297^b^		
Diabetic nephropathy	35.17	0.749		
STZ+ Caffeic acid	19.43	0.429^b^		

^a^p < 0.05 compared to non diabetic control. ^b^p < 0.05 compared to STZ induced DN control group.

### Effect of caffeic acid on the renal function of HFD-STZ induced diabetic rats

**Figure 2:**
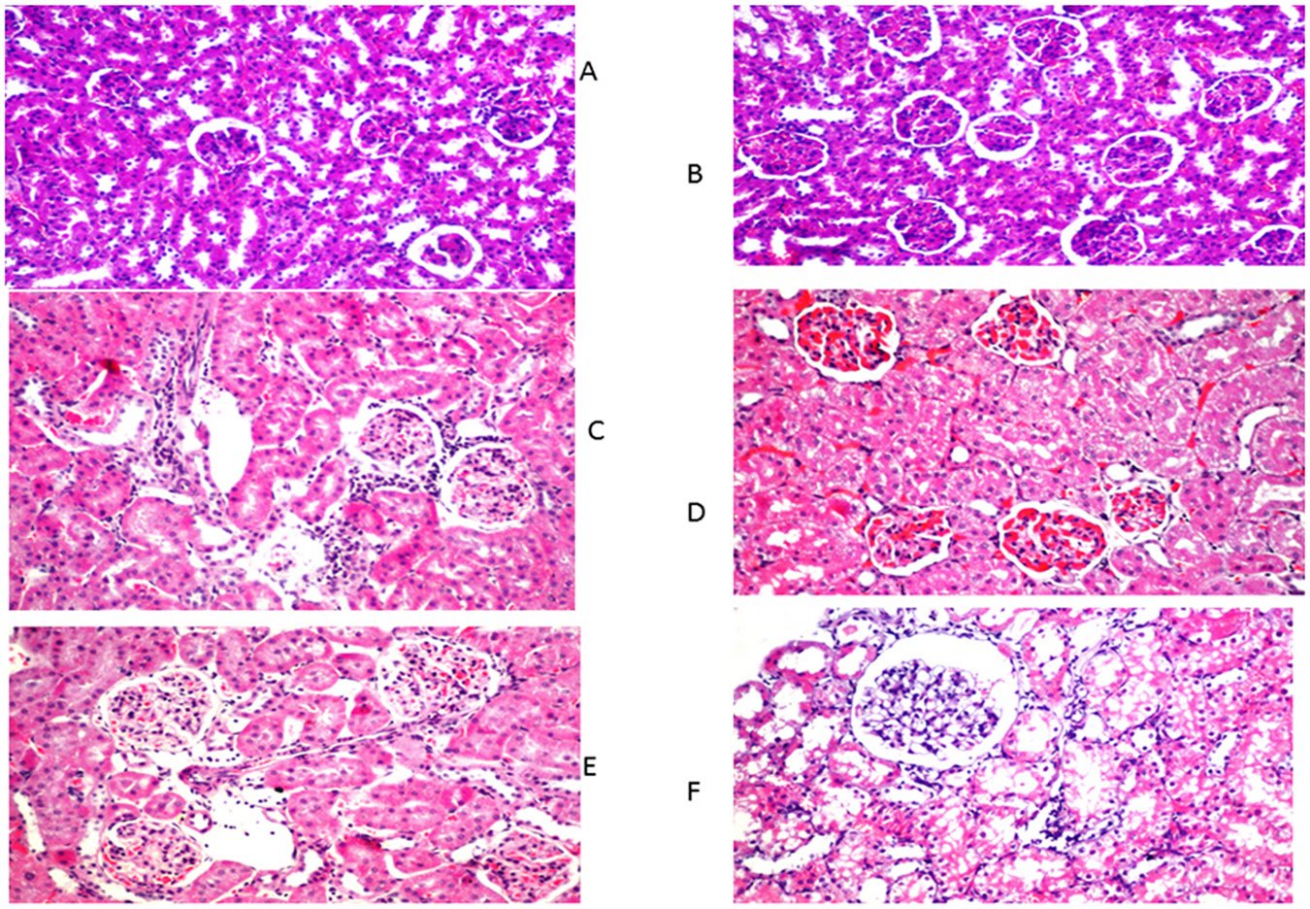
Histopathalogical studies of kidney. (**A**) Control; (**B**) Naive; (**C**) Diabetes mellitus group; (**D**) Diabetic nephropathy group; (**E**) caffeic acid prophylactic group; (**F**) caffeic acid treated group. H&E X 400.

Urinary albumin, urinary creatinine, serum creatinine, blood urea nitrogen (BUN), urea and creatinine clearance in DN group were increased compared to NC group. Dose-response effects of CA on creatinine clearence in HFD-STZ-rats was performed. Increasing the dose of CA resulted in an improvement of creatinine clearence. Thus, we found that CA had a pronounced dose-dependent effect on improvement of creatinine clearance (Fig. 2) and 40–50 mg/kg of CA were the most effective doses for improving creatinine clearence in HFD-STZ rats.

The ameliorating effect of caffeic acid on renal function parameters was evident both in the treatment and prophylactic groups, (Table 1).

### Effect of caffeic acid on renal histology and autophagy induction in HFD-STZ induced diabetic rats

**Figure 3:**
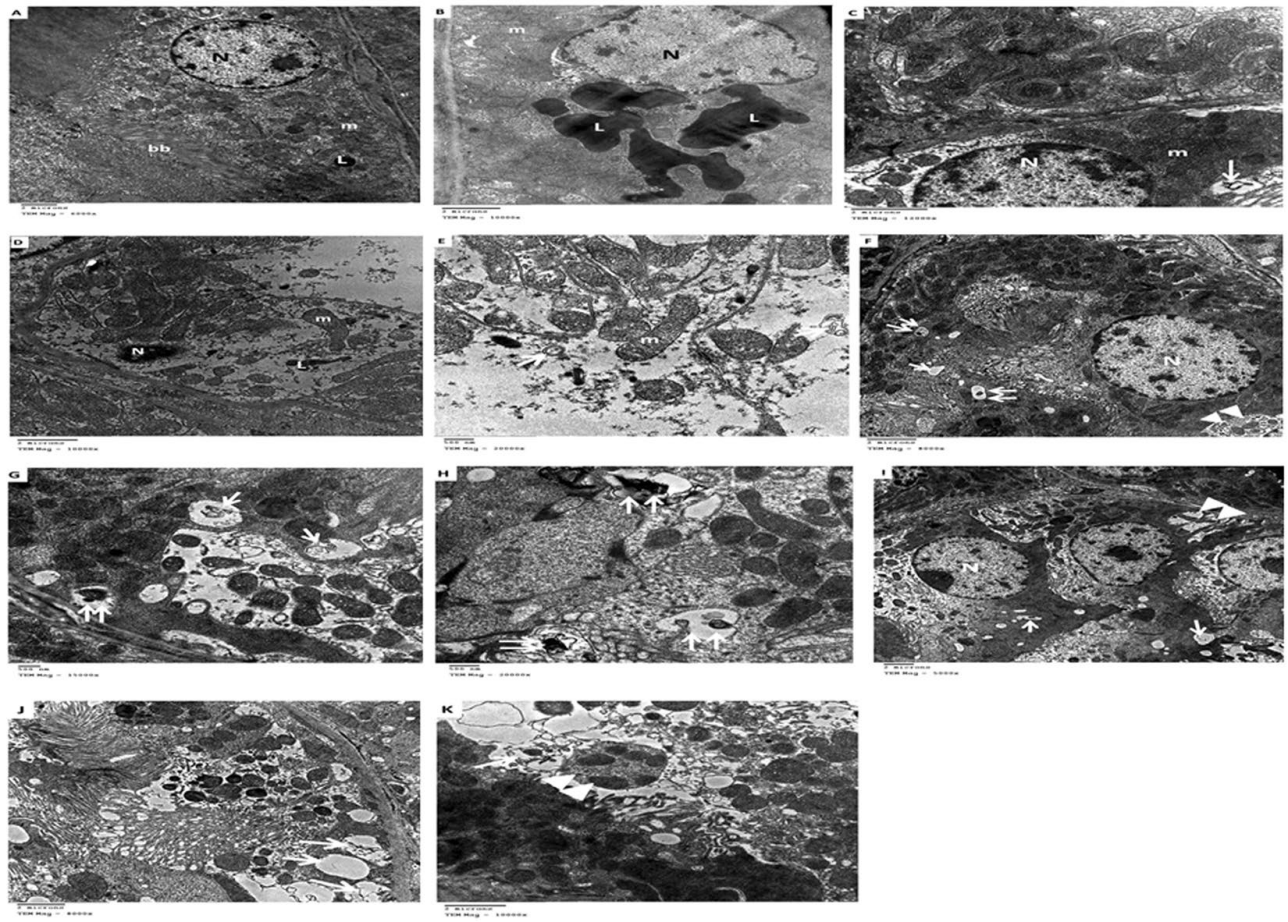
Transmission electron micrograph of renal tubules. (**A**) Control group: Normal structures of the nucleus, mitochondria with few scattered electron-dense lysosomes and intact bruch border were noted. (**B** and **C**) diabetic rats: accumulation of huge electron-dense lysosomes and few autophagic vacuoles were detected. (**D** and **E**) diabetic nephropathy group: apoptotic nucleus, partially degraded cytoplasm and elongated mitochondria. Also, double membrane of the autophagosome was clearly seen containing cytoplasmic debris. (**F**–**H**) treated diabetic rats: cytoplasm showed multiple scattered autophagosomes, autophagosomes with lysosomes (vacuoles containing electron dense material), and mitophagy (an autophagosome containing an undigested mitochondrion), indicative of autophagic activity. (**I**–**K**) diabetic prophylactic rats: numerous autophagosomes with degenerated membrane cellular debris and mitophagy **L**, lysosomes; **N**, nucleus; **bb**, bruch border; **m**, mitochondria; [↑], autophagosomes; [↑↑], autophagosomes with lysosomes; ▴▴, mitophagy.

Parallel to the development of DM in HFD-STZ treated rats, histopathological examination of H&E stained kidney tissues by light microscopy revealed a focal inflammatory cells infiltration as well as detection of aggregation in-between the degenerated tubules and glomeruli at the cortex in diabetic group. Moreover, experimentally induced DKD group showed severe congestion in the glomeruli associated with swelling in the tubular lining epithelium at the cortex. Furthermore, prophylactic administration of caffeic acid in HFD-STZ induced diabetic rats showed mild congestion in the glomeruli associated with swelling in the lining tubular epithelium. Lastly treatment with caffeic acid in HFD-STZ induced diabetic rats resulted in glomerular vacuolization in the lining endothelium of the tufts associated with degeneration in the tubular lining epithelium at the cortex. All in all, prophylactic and caffeic acid treated group, significantly ameliorated the renal changes with extensive vacuolation(Fig. 2A–E). To visualize autophagy induction by caffeic acid in DKD, TEM was performed. Electron micrographs of autophagosomes was demonstrated in figure (Fig. 3A–K). Rare autophagic vacuoles were detected in the tubular cells of the control and diabetic rats. However, an increased level of autophagy was observed in the groups treated with caffeic and prophylactic group. Autophagic vacuoles including autophagosomes and autophagolysosomes were markedly increased in the proximal tubular cells. Double membrane vacuoles containing electron-dense material, degenerating cytoplasmic organelles and cytosol, mitochondria with loss of visible cristae were frequently observed in the tubules.

### Effect of caffeic acid on the expression of autophagy transcripts in DKD

At the end of the 12 weeks, *RB1CC1*, *ATG12*, *MAP1LC3B* mRNA expression were estimated in the kidney of the 6 groups and were downregulated in the DKD group compared to the naïve group (3.8, 5.2 and 13 fold, respectively).

**Figure 4:**
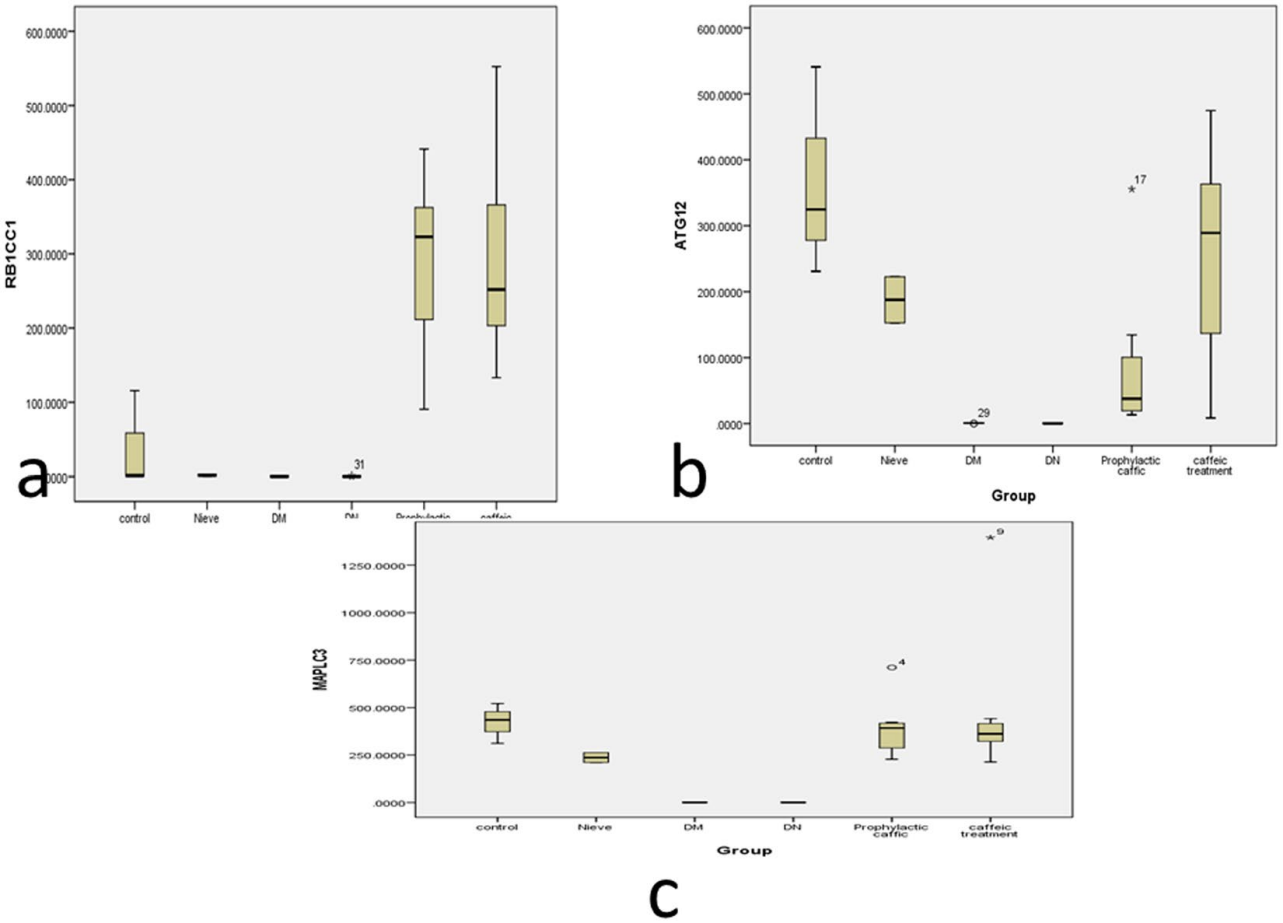
*RB1CC1, ATG12, MAP1LC3B* expression in Kidney tissue at the end of the 12 weeks in the Control, Naive, Diabetes mellitus group, Diabetic nephropathy group, caffeic acid Prophylactic group, caffeic acid Treated group. (**A**) *RB1CC1* mRNA; (**B**) *ATG12* mRNA; (**C**) *MAP1LC3B*. Data are presented as fold change, where *indicates P < 0.05 compared to the control group (One way ANOVA followed by Bonferroni’s multiple comparison test), N = 8/group.

Caffeic acid administration in the treated and prophylactic groups resulted in a significant induction of renal *RB1CC1*, *ATG12*, *MAP1LC3B* mRNA expression, compared to the control group [F = 6.3,4.9 and 4.7, respectively, at p < 0.01]. However, we could not find any significant difference between the treated and prophylactic groups (Table 2 and Fig. 4a–c).

**Table 2:** Differential Expression of Autophagy transcript among different groups of animal model.

	Mean	Std. Error	F	Significance
**ATG12**				
control	365.540000	91.7983929	4.960	0.001
Nieve	187.699700	35.0997000		
DM	0.653364	0.1643958		
DN	0.518168	0.2456460		
Prophylactic caffeic	92.360886	46.6909842		
caffeic treatment	253.094229	66.1801127		
**MAPLC3**				
control	422.599900	60.8979453	4.797	0.001
Nieve	236.678550	25.6485500		
DM	0.542120	0.0963318		
DN	0.511700	0.1984661		
Prophylactic caffeic	391.589014	60.9800118		
caffeic treatment	492.281086	152.8860110		
**RB1CC1**				
control	39.311867	38.1394967	6.379	0.000
Nieve	1.853000	0.7741000		
DM	0.313524	0.1130870		
DN	0.306620	0.1765692		
Prophylactic caffeic	286.109286	45.7528180		
caffeic treatment	296.567486	54.8583762

### Effect of caffeic acid on the renal expression of miR-133b, −342,30a in DKD

At the end of the 12 weeks, miR-133b, -342, 30a expression were estimated in the kidney of the 6 groups and were up-regulated in the DKD group compared to the naïve group (1900, 175 and 125 fold, respectively).

**Figure 5:**
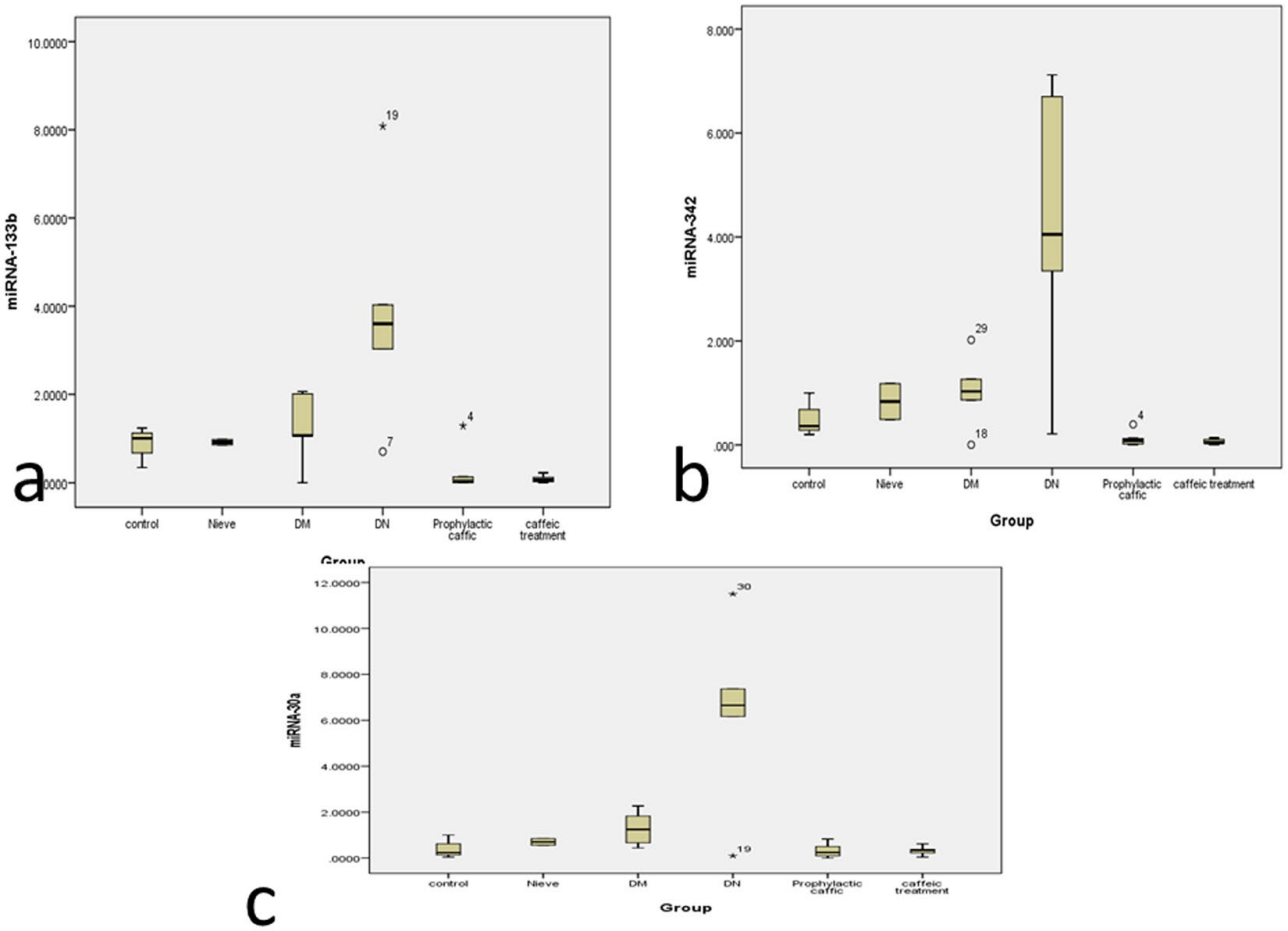
miR-133b, -342, 30a expression in Kidney tissue at the end of the 12 weeks in the Control, naïve, Diabetes mellitus group, Diabetic nephropathy group, caffeic acid Prophylactic group, caffeic acid Treated group. (**A**) miR-133b; (**B**) miR-342; (**C**) miR-30a. Data are presented as fold change, where *indicates P < 0.05 compared to the control group (One way ANOVA followed by Bonferroni’s multiple comparison test), N = 8/group.

Caffeic acid administration in treated and prophylactic groups resulted in a significant down regulation of renal miR-133b, -342, 30a expression, compared to the control group [F = 6.7,7.896 and 7.814, respectively at p < 0.01]. Notably, there was no significant difference between the treated and prophylactic groups (Table 3 and Fig. 5a–c). Of note, there was a significant negative correlation between the chosen DN-characteristic miRNAs and their corresponding autophagy target genes(p < 0.01) among all the groups of the study (Table 4).

**Table 3:** miRNA Expression among different groups of animal model.

	Mean	Std. Error	F	Significance
**miRNA-133b**				
control	0.857467	0.2681801	6.769	0.0001
Nieve	0.913150	0.0578500		
DM	1.239480	0.3786406		
DN	3.888120	1.1955332		
Prophylactic caffeic	0.225943	0.1773867		
caffeic treatment	0.081271	0.0306317		
**miRNA-342**				
control	0.52057	0.244219	7.896	0.0001
Nieve	0.83430	0.344400		
DM	1.03610	0.325210		
DN	4.28552	1.253075		
Prophylactic caffeic	0.10760	0.051428		
caffeic treatment	0.06489	0.019441		
**miRNA-30a**				
control	0.424800	0.2929798	7.814	0.0001
Nieve	0.701900	0.1481000		
DM	1.288600	0.3440706		
DN	6.355700	1.8278039		
Prophylactic caffeic	0.320486	0.1147793		
caffeic treatment	0.310000	0.0691868

**Table 4:** Correlation between the selected miRNAs and autophagy transcript among the investigated groups of rats.

Correlations						
	miRNA-133b	miRNA-342	miRNA-30a	ATG12	MAPLC3	RB1CC1
**miRNA-133b**						
Correlation Coefficient	1.000	0.893^**^	0.546^**^	−0.483^**^	−0.425^**^	−0.543^**^
Sig. (2-tailed)		0.000	0.001	0.003	0.010	0.001
**miRNA-342**						
Correlation Coefficient	0.893^**^	1.000	0.685^**^	−0.467^**^	−0.517^**^	−0.569^**^
Sig. (2-tailed)	0.000		0.000	0.004	0.001	0.000
**miRNA-30a**						
Correlation Coefficient	0.546^**^	0.685^**^	1.000	−0.442^**^	−0.468^**^	−0.375^*^
Sig. (2-tailed)	0.001	0.000		0.007	0.004	0.024
**ATG12**						
Correlation Coefficient	−0.483^**^	−0.467^**^	−0.442^**^	1.000	0.547^**^	0.530^**^
Sig. (2-tailed)	0.003	0.004	0.007		0.001	0.001
**MAPLC3**						
Correlation Coefficient	−0.425^**^	−0.517^**^	−0.468^*^	0.547^**^	1.000	0.754^**^
Sig. (2-tailed)	0.010	0.001	0.004	0.001		0.000
**RB1CC1**						
Correlation Coefficient	−0.543^**^	−0.569^**^	−0.375^*^	0.530^**^	0.754^**^	1.000
Sig. (2-tailed)	0.001	0.000	0.024	0.001	0.000	

**Correlation is significant at the 0.01 level (2-tailed). *Correlation is significant at the 0.05 level (2-tailed). ***Indicates P < 0.001, Unpaired, two tailed t test, N = 8/group. r:Spearman correlation coefficient.

## Discussion

Until the current study, whether caffeic acid exerted any curative or prophylactic effect in DN was unknown. To address this question, we investigated the effect of caffeic acid on urinary albumin excretion in HFD-STZ induced diabetic rats. Obesity was induced in rats with high-fat diet and streptozotocin injection, which affects islet β-cells and results in insulin resistance^12, 13^. Urinary albumin has been used as a predictive biomarker for prognosis of DN. Furthermore, the reduction in urinary albumin in DN reportedly were correlated with renal protection^14^. Herein, we show that DN caused significant increases in the urinary albuminuria, whereas caffeic acid suppressed this effect.

Caffeic acid treatment and prophylaxis markedly increased *RB1CC1*, ATG12, *MAP1LC3B* mRNA expression in diabetic kidneys and decreased congestion with marked vacuolation in diabetic glomeruli. We used transmission electron microscopy (TEM) to monitor the appearance of autophagosomes. As shown in Fig. 5A, no obvious autophagic vacuoles were found in control with numerous autophagic vacuoles appeared in caffeic acid treated and prophylactic group. In addition, Caffeic acid inhibited miR-133b, -342, 30a expression (epigenetic regulators of the chosen autophagy genes) in diabetic nephropathy rats. Our findings suggested that caffeic acid resulted in marked induction of autophagy in diabetic kidneys with potential curative and preventative effect. In agreement with previous studies that addressed the role of caffeic acid in improving glucose utilization in insulin-resistant mouse hepatocytes^15^. Remarkably, caffeic acid phenethyl ester, a phytochemical extraxt of propolis was found to have antidiabetic effect and improve renal function tests in a rat model with renal tubular damage and oxidative stress^16, 17^. Moreover, previous studies explored the role of caffeic acid and its derivative in induction of autophagy^10, 18^.

Accumulating evidences indicate that change in the nutrient-sensing paths in diabetic states possibly will alter the autophagic response stimulated by cellular stress, which could subsequently result in diabetic nephropathy^19^. Impaired autophagy lead to accumulation of p62/SQSTM1 protein in proximal tubular cells^20^, activation of the mTOR pathway^21^ and inactivation of AMPK^22^. Modulation of the autophagy pathway has a great impact on development of a new nephro protective and therapeutic option^23^. Caffeic acid triggered induction of AMPK, class III PI3-kinase and the autophagic response in tumor^11, 28^.

Autophagy involves a series of dynamic membrane rearrangements controlled by a set of ATG proteins^24^. RB1-inducible coiled-coil protein 1(RB1CC1 or ATG 17), which are needed for phagophore formation and initiation of autophagy^25, 26^. Microtubule-associated proteins 1A/1B light chain 3B is a central gene in the autophagy pathway where it functions in autophagosome membrane expansion and fusion events and have structural homology with ubiquitin^27^. ATG12, forms the ATG12–ATG5–ATG1 complex involved in autophagosome maturation^28^. Zahng *et al*., reported that, erlotinib increased renal autophagy, as indicated by altered expression and activity of ATG12, and LC3A II, in diabetic mice^29^. Fang and his colleagues demonstrated that the expression of autophagy related proteins such as Beclin-1, ATG12-ATG5 and LC3-II was markedly inhibited in DKD^30^. Recent evidence showed that induction of autophagy may be linked to maintaining renal homeostasis in diabetic kidney^31^. In the light of our results, it seems that caffeic acid might improve diabetic nephropathy through the restoration of autophagy activity in diabetic kidneys.

Because miRNAs are recently linked to regulation of autophagy pathway^32^, we applied combined bioinformatics analysis to retrieve DN related autophagy genes and their miRNA regulators. Accordingly, miR-133b, -342 and -30a target the above mentioned autophagy genes and were previously reported by our research group as urine markers for DN^33^. miR-30a inhibits autophagy by selectively down regulating ATG5 and Beclin 1 expression^34^. Targeting miR-30a, induces autophagy in response to imatinib treatment in chronic myeloid leukemia^35^. miR-30a and miR-34a play their key roles in the regulation of autophagy pathway such as PI3KCI/Akt/mTORC1 signaling pathway, and Ras-Raf-MAPK cascade^36^. Interestingly, miR-133b induced autophagic cell death in colon cancer cells^37^. Vitamin E-based therapy for hyperglycemia & T2DM triggers the expression of AMPK via regulation of miRNA-133b^38^. Moreover, miR-342-5p is coupled to the antiviral IFN response^39^ which in turn linked to autophagy.

Our Experimental model revealed that treatment with caffeic acid suppressed the expression of these miRNAs with subsequent induction of autophay which ameliorated glomerular changes, albuminuria with reducing blood glucose levels in HFD-STZ -induced diabetic rats. We hypothesize that caffeic acid seems to trigger AMPK signaling, PIK3 pathway via regulation of miR-30a,-342,-133b which in turn induces autophagy that ameliorates diabetic nephropathy.

## Conclusion

We adopt alternative strategies for better management of DN with the interest in the search of new drugs from natural sources and determining their mechanism of action which will be of great value in developing countries with limited resources and high incidence rates of diabetes mellitus. Also, there is a profitable concern in the advancement of tools to suppress or stimulate miRNA expression associated with autophagy markers in vivo as an advance in the management of diabetes complications.

## Material and Methods

### Animal Experiments

All procedures for the care and use of laboratory animals were approved by the Institutional Animal Ethics Committee for Ain Shams University, Faculty of Medicine. All the methods were performed in accordance with the relevant Ethical guidelines and regulations (Ethical committee approval no FWA 000017585). Male Wistar rats (weighing 250–300 g) were purchased from National Research Institute (Cairo, Egypt) and were accommodated in an animal house with temperature (22 ± 1C) and lighting (12 h light– dark cycle) control. Before the start of the experimental work, an adaptation one week during which rates were administered vehicle (tap water).

### Experimental protocol

**Figure 6:**
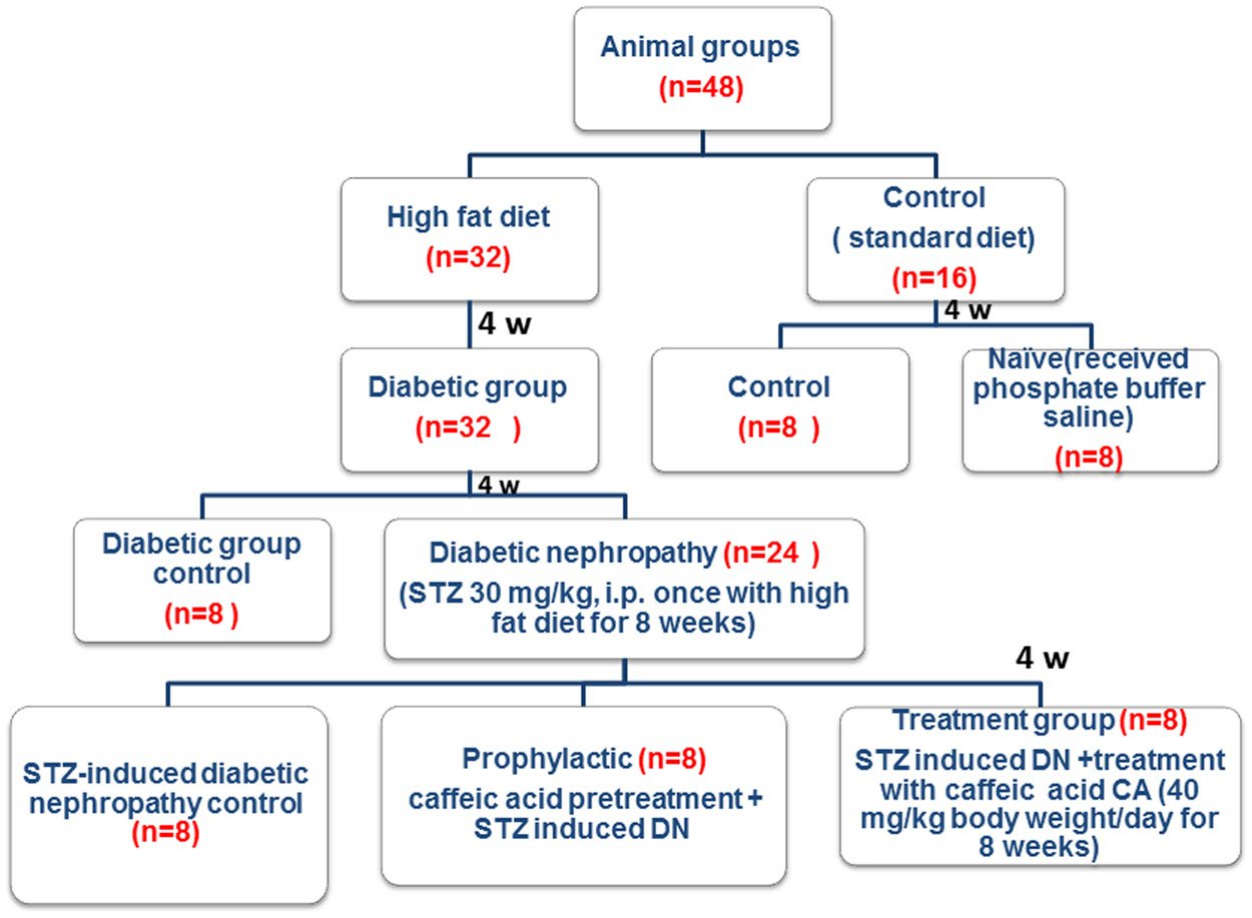
Flow chart of animal groups.

**Figure 7:**
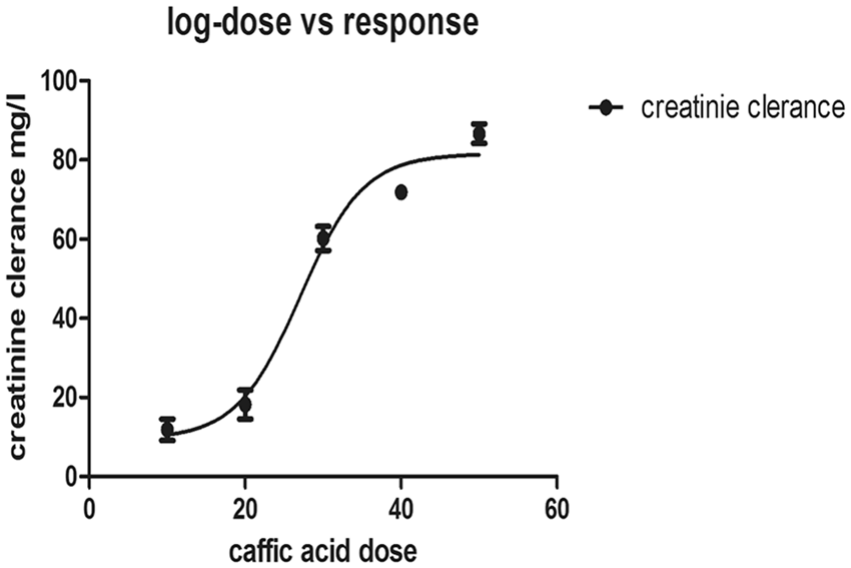
Dose-response effects of Caffeic acid on creatinine clearence level in HFD-STZ-rats. STZ rats were administered Caffeic acid different doses range from (10–50 mg/kg). Creatinine clearence was determined after 4 weeks of caffeic acid treatment. The data are expressed as the means ± SEM.

Caffeic acid and STZ were purchased from Sigma Aldrich^40^. Forty eight male Wistar rats weighing 250–300 g were divided into six groups (Fig. 6), 8 animals each as shown in Fig. 1. After one week adaptation, rats were splitted into a high-fat group (32 rats) which obtained a high-fat diet for four weeks and a normal age-matched control group (16 rats) which received a standard diet and subdivided into control and naïve groups. All high fat group after 4 weeks had received STZ 30 mg/kg. i.v. once with high fat diet for another 4 weeks). HFD-STZ developed type II DM with fasting blood glucose above 16.7 mmol/L. Afterwards, they were haphazardly divided into two groups: diabetic control group (8 rats), DKD model group, 24 rats (They were further subdivided into three groups: diabetic nephropathy control group (8 rats); herbal extract caffeic acid (CA) treated group, 8 rats (CA 40 mg/kg body weight/day orally for 4 weeks) and pretreated group: 8 rats (received the herbal extract CA after induction of diabetes for 4 weeks). CA was dissolved in cold water and administrated via intra-gastric gavage (i.g.) one time daily for twelve weeks according to Jayanthi *et al*.^40^ and Dhungyal *et al*.^41^ (Fig. 1). To study the effectiveness of CA at different doses in modulating renal function, creatinine clearance was estimated after CA treatment period in the HFD- STZ rats (Fig. 7).

### Induction of DKD

Type 2 diabetes mellitus was provoked according to Zhang *et al*.^42, 43^. In the normal group, rats were fed a standard chow diet of a total kcal value of 20 kJ/kg (52% carbohydrate, 20% protein, 5% fat), while diabetic group rates were fed a high-fat diet of a total kcal value of 40 kJ/kg (45% carbohydrate, 22% protein, 20% fat). The two groups were kept on their diets for 8 weeks duration. In the 4^th^ week, a single low dose of STZ (30 mg/kg, dissolved in 0.1 M sodium citrate buffer at pH 4.4) was injected into each rat of the diabetic group intraperitoneally.

After the STZ injection, rats obtained drinking water containing sucrose (15 g/L) for 48 hours, to reduce early death due to insulin discharge from partially injured pancreatic islets. Seventy two hours later, rats were checked for hyperglycemia and those with FBS more than 250 mg/dL were included in studies of diabetic nephropathy. Diabetic rats received long-acting insulin (2–4 U/rat) via S.C injection to maintain blood glucose levels in a desirable range (300 mg/dL) and also to prevent subsequent development of ketonuria^44^. Nephropathy was noted in rats (4–8 weeks) after the administration of STZ and was assessed in terms of significant increase in proteinuria, serum creatinine, blood urea nitrogen (BUN), extracellular matrix deposition and thickening of glomerular basement membrane. At the end of the eight-week pretreated and treated, rats were sacrificed using an intraperitoneal injection of sodium pentobarbital (50 mg kg^−1^). Blood samples and kidney were collected for biochemical, histopathological analyses and TEM. A 24-h urine was collected on the day before scarification.

### Metabolic parameters, urinary albumin excretion, and renal function analysis

Fasting blood sugar(FBG) was measured using the glucose oxidase method. Serum total cholesterol, LDL, HDL, Triglycerides, creatinine, and urine creatinine were detected by automated clinical chemistry analyzer (Olympus- 2000, Tokyo, Japan). Samples from rat kidneys were snap frozen at −80 °C for further RNA extraction, and another samples were processed for histopathological examination and TEM.

### Histopathological Examination

Kidneys were fixed (10% neutral buffered formalin (NBF)), paraffin embedded, cut into 4 μm sections and then stained with Hematoxylin and Eosin to be examined at 400 magnification by 2 independent histopathologists.

### Transmission electron microscopy (TEM)

Kidney tissue samples were fixed in glutaraldehyde and osmium tetroxide, washed with PBS& dehydrated. After exchange through acetone, the samples were later embedded in Epon 812. The kidney tissue were made into ultra-thin (70–80 nm) after observation and positioning, and were double stained following standard methods. All of the kidney samples were examined using TEM.

### Quantitative real-time PCR analysis for measurement of miRNAs and autophagy transcripts

We have retrieved a set of 3 DN-characteristic miRNAs (miR-133b, miR-342 and miR-30a) based on previous microarray studies such as miro-Ontology database (available at http://ferrolab.dmi.unict.it/miro/), miRWalk database distinguished DKD from other diseases. These 3 miRNAs were chosen related to diabetic nephropathy and targeting autophagy genes(RB 1-inducible coiled coil protein (RB1CC1), Microtubule-associated proteins 1A/1B light chain 3(MAP1LC3B), Autophagy related gene (ATG-12)].

Total RNA, including small RNA species, was extracted from kidney using miRNeasy® Mini kit (Qiagen, Germany), according to the manufacturer’s recommendations. Total RNA concentration was measured by measuring absorbance at 260 nm using an Ultraspec 1000, UV/visible spectrophotometer (Amersham Pharmacia Biotech, Cambridge, England). Afterwards, A260/A280 and A260/A230 ratios were determined. A260/A280 ratio must be between 1.8 and 2^45^.

Afterwards, 500 ng total RNA from kidney was changed to cDNA by miScript II RT Kit (Qiagen, Valencia, CA) using miScript HiSpec buffer which was used in real-time PCR analysis using a miScript primer assay and the miScript SYBR Green Kit. The miScript Universal Primer (reverse primer) and QuantiTect SYBR Green PCR Master Mix, respectively were used as per manufacturer’s protocol to detect 3 miRs (miR-133b, miR-342, miR-30a) and 3 autophagy genes(*RB1CC1, ATG12* and *MAP1LC3B*), respectively. RNA quality control and housekeeping gene (RNU6-2, *GADPH*) were also included in the assay for miRNA and autophagy gene expression, respectively. The qPCR tubes were run on a Step One Plus™ System (ABi). The primer of selected miRNAs, autophagy transcript and endogenous control were purchased from Qiagen. Data Analysis were done by using the ΔΔCT method of relative quantification^46^ on a Light- cycler, software v2.2.2 (StepOne™ Software). Data were presented as fold change in expression and were calculated as 2^(−ΔΔCT)^. Where ΔCT = CT target gene – AVG CT reference gene and ΔΔCT = ΔCT (sample 2) − ΔCT (sample 1) where sample 1 is the control sample and sample 2 is the experimental sample.

### Statistical tests

Data are presented as mean ± S.D. Statistical differences between the groups were estimated by one-way analysis of variance (ANOVA) followed by Bonferroni’s post-tests. A p-Value < 0.05 was considered significant. Statistical analyses carried out using Graph Pad Prism (GraphPad software) and with SPSS version 21.0 (SPSS, Chicago, IL, USA).

## References

[1] Nasri H (2013). On the occasion of the world diabetes day 2013; diabetes education and prevention; a nephrology point of view. J Renal Inj Prev..

[2] Tikoo K, Tripathi DN, Kabra DG, Sharma V, Gaikwad AB (2007). Intermittent fasting prevents the progression of type I diabetic nephropathy in rats and changes the expression of Sir2 and p53. FEBS Lett..

[3] Fang L (2013). Autophagy attenuates diabetic glomerular damage through protection of hyperglycemia-induced podocyte injury. PLoS One..

[4] Periyasamy-Thandavan S (2008). Autophagy is cytoprotective during cisplatin injury of renal proximal tubular cells. Kidney Int..

[5] Zhou YF, Fu JL, Guan YF (2012). MicroRNAs and diabetic nephropathy. Sheng Li KeXue Jin Zhan..

[6] Jegga AG, Schneider L, Ouyang X, Zhang J (2011). “Systemsbiology of the autophagy-lysosomal pathway”. Autophagy.

[7] Lei Z, Li B, Yangetal Z (2009). “Regulation of HIF-1 and VEGF by miR-20b tunes tumor cells to adapt to the alteration of oxygen concentration”. PLoS ONE.

[8] Ahmed ABA, Rao ASA, Rao MV (2010). *In vitro* callus and *in vivo* leaf extract of Gymnemasylvestre stimulate β-cells regeneration and anti-diabetic activity in Wistar rats. Phytomedicine.

[9] Wang ZQ (2011). An extract of Artemisia dracunculus L. enhances insulin receptor signaling and modulates gene expression in skeletal muscle in KK-Ay mice. J. Nutr. Biochem..

[10] Vijayakurup V (2014). Phenethyl caffeate benzoxanthene lignan is a derivative of caffeic acid phenethyl ester that induces bystander autophagy in Wi Dr cells. Mol Biol Rep..

[11] Mamal E, Basar M, Uzun H, Seckin I (2012). Caffeic Acid Phenethyl Ester Prevents Mesengial Cell Apoptosis by Suppressing p38MAPK Signal. J.Cytol. Histol..

[12] Liu XX (2014). Adiponectin is expressed in the pancreas of high-fat-diet-fed mice and protects pancreatic endothelial function during thedevelopment of type 2 diabetes. Diabetes Metab.

[13] Huang Y, Chen E, Chen Y (2010). Establishment a rat model for non-insulin-dependent diabetes mellitus induced by streptozotocin in combination with high-sugar and high-fat diets. J Practical Med.

[14] Satirapoj B (2012). Nephropathy in diabetes. Advances in Experimental Medicine and Biology.

[15] Huang DW, Shen SC (2012). Caffeic acid and cinnamic acid ameliorate glucose metabolism via modulating glycogenesis and gluconeogenesis in insulin-resistant mouse hepatocytes. Journal of Functional Foods.

[16] Oktem F (2005). Lithium-induced renal toxicity in rats: protection by a novel antioxidant caffeic acid phenethyl ester. Mol. Cell. Biochem..

[17] Weng (2012). “Caffeic Acid Phenylethyl Amide Protects against the Metabolic Consequences in Diabetes Mellitus Induced by Diet and Streptozocin”. Evidence-Based Complementary and Alternative Medicine.

[18] Yu SH (2011). Inhibition of AMPK-associated autophagy enhances caffeic acid phenethyl ester-induced cell death in C6 glioma cells. Planta Med..

[19] Kimura T (2011). Autophagy protects the proximal tubule from degeneration and acute ischemic injury. J Am Soc. Nephrol..

[20] Yamahara K (2013). Obesity-mediated autophagy insufficiency exacerbates proteinuria-induced tubulointerstitial lesions. J Am Soc. Nephrol..

[21] Zoncu R, Efeyan A, Sabatini DM (2011). mTOR: from growth signal integration to cancer, diabetes and ageing. Nat Rev Mol Cell Biol..

[22] Kitada M, Kume S, Imaizumi N, Koya D (2011). Resveratrol improves oxidative stress and protects against diabetic nephropathy through normalization of Mn-SOD dysfunction in AMPK/SIRT1-independent pathway. Diabetes..

[23] Kume S, Thomas MC, Koya D (2012). Nutrient sensing, autophagy, and diabetic nephropathy. Diabetes..

[24] Hartleben B (2010). Autophagy influences glomerular disease susceptibility and maintains podocyte homeostasis in aging mice. J Clin Invest..

[25] Khan S (2016). Role of autophagy and histone deacetylases in diabetic nephropathy: Current status and future perspectives. Genes & Diseases.

[26] Yao J (2015). Deletion of autophagy inducer RB1CC1 results in degeneration of the retinal pigment epithelium. Autophagy..

[27] Weidberg H (2011). “LC3 and GATE-16 N termini mediate membrane fusion processes required for autophagosome biogenesis”. Developmental Cell.

[28] Glick D, Barth S, Macleod KF (2010). Autophagy: cellular and molecular mechanisms. J Pathol..

[29] Zhang MZ, Wang Y, Paueksakon P, Harris RC (2014). Epidermal growth factor receptor inhibition slows progression of diabetic nephropathy in association with a decrease in endoplasmic reticulum stress and an increase in autophagy. Diabetes..

[30] Fang L (2013). Autophagy Attenuates Diabetic Glomerular Damage through Protection of Hyperglycemia-Induced Podocyte Injury. PLoS ONE.

[31] Chang CC (2011). Resveratrol retards progression of diabetic nephropathy through modulations of oxidative stress, proinflammatory cytokines, and AMP-activated protein kinase. J Biomed Sci..

[32] Frankel LB, Lund AH (2012). MicroRNA regulation of autophagy. Carcinogenesis..

[33] Eissa S, Matboli M, Bekhet MM (2016). Clinical verification of a novel urinary microRNA panal: 133b, −342 and −30 as biomarkers for diabetic nephropathy identified by bioinformatics analysis. Biomedicine& pharmacotherapy..

[34] Yu Y (2012). microRNA 30A promotes autophagy in response to cancer therapy. Autophagy..

[35] Yu Y (2012). Targeting microRNA-30a-mediated autophagy enhances imatinib activity against human chronic myeloid leukemia cells. Leukemia..

[36] Liu B, Wen X, Cheng Y (2013). Survival or death: disequilibrating the oncogenic and tumor suppressive autophagy in cancer. Cell Death and Disease.

[37] Taniguchi K (2016). PTBP1-associated microRNA-1 and -133b suppress the Warburg effect in colorectal tumors. Oncotarget..

[38] Boominathan., Vitamin-based therapy for Hyperglycemia & T2DM: Vitamin-E (Tocopherol) increases the expression of AMPK, decreases gluconeogenesis and fasting glucose level via up regulation of its target gene, 8/June/2015, 6.56 am, Genome-2-Bio-Medicine Discovery center (GBMD), http://genomediscovery.org, accessed on 14-3-2016.

[39] Robertson KA (2016). An Interferon Regulated MicroRNA Provides Broad Cell-Intrinsic Antiviral Immunity through Multihit Host-Directed Targeting of the Sterol Pathway. PLoS Biol..

[40] Jayanthi R, Subash P (2010). Antioxidant Effect of Caffeic Acid on Oxytetracycline Induced Lipid Peroxidation in Albino Rats. Ind J Clin Biochem.

[41] Dhungyal B, Koirala P, Sharma C, Jha DK (2014). Caffeic Acid - A Potent Phytocompound against Diabetes Mellitus A Review. SMU Medical Journal.

[42] Zhang M, Lv XY, Li J, Xu ZG, Chen L (2008). The characterization of high-fat diet and multiple low-dose streptozotocin induced type 2 diabetes rat model. Experimental Diabetes Research..

[43] Liu Z (2013). Antidiabetic effects of malonylginsenosides from Panax ginseng on type 2 diabetic rats induced by high-fat diet and streptozotocin. J. Ethnopharmacol..

[44] Graham ML, Janecek JL, Kittredge JA, Hering BJ, Schuurman HJ (2011). The streptozotocin-induced diabetic nude mouse model: differences between animals from different sources. Comp Med..

[45] Fleige S, Pfaffl MW (2006). RNA integrity and the effect on the real-time qRT-PCR performance. Molecular aspects of medicine.

[46] Livak KJ, Schmittgen TD (2001). Analysis of relative gene expression data using real-time quantitative PCR and the 2(-Delta Delta C(T)) Method. Methods (San Diego, Calif)..

